# Anti-Osteoporotic Effect of Morroniside on Osteoblast and Osteoclast Differentiation In Vitro and Ovariectomized Mice In Vivo

**DOI:** 10.3390/ijms221910642

**Published:** 2021-09-30

**Authors:** Chang Gun Lee, Jeonghyun Kim, Seung Hee Yun, Seokjin Hwang, Hyoju Jeon, Eunkuk Park, Seon-Yong Jeong

**Affiliations:** 1 Department of Medical Genetics, Ajou University School of Medicine, Suwon 16499, Korea; dangsunsang@naver.com (C.G.L.); danbi37kjh@hanmail.net (J.K.); yun41101@ajou.ac.kr (S.H.Y.); tjrwlshh@naver.com (S.H.); wjsgywn0315@ajou.ac.kr (H.J.); 2 Department of Biomedical Sciences, Ajou University Graduate School of Medicine, Suwon 16499, Korea

**Keywords:** bone remodeling, morroniside, primary cultured osteoblasts, primary cultured osteoclasts, ovariectomized mice

## Abstract

Bone remodeling is a continuous process of bone synthesis and destruction that is regulated by osteoblasts and osteoclasts. Here, we investigated the anti-osteoporotic effects of morroniside in mouse preosteoblast MC3T3-E1 cells and mouse primary cultured osteoblasts and osteoclasts in vitro and ovariectomy (OVX)-induced mouse osteoporosis in vivo. Morroniside treatment enhanced alkaline phosphatase activity and positively stained cells via upregulation of osteoblastogenesis-associated genes in MC3T3-E1 cell lines and primary cultured osteoblasts. However, morroniside inhibited tartrate-resistant acid phosphatase activity and TRAP-stained multinucleated positive cells via downregulation of osteoclast-mediated genes in primary cultured monocytes. In the osteoporotic animal model, ovariectomized (OVX) mice were administered morroniside (2 or 10 mg/kg/day) for 12 weeks. Morroniside prevented OVX-induced bone mineral density (BMD) loss and reduced bone structural compartment loss in the micro-CT images. Taken together, morroniside promoted increased osteoblast differentiation and decreased osteoclast differentiation in cells, and consequently inhibited OVX-induced osteoporotic pathogenesis in mice. This study suggests that morroniside may be a potent therapeutic single compound for the prevention of osteoporosis.

## 1. Introduction

Osteoporosis is a common skeletal disorder characterized by bone mineral density (BMD) loss caused by the dysregulation of bone homeostasis, including bone resorption by osteoclasts and bone formation by osteoblasts [1]. The imbalance between the two orchestrated processes leads to weak and brittle bone, increasing the risk of bone diseases such as osteoporosis [2]. Osteoporosis is caused by multiple factors, such as hormone defects, genetic ablation, and environmental agents [3]. Recent pharmacological medications for osteoporosis treatment have focused on inhibiting either the cause or process of osteoporosis pathogenesis [4]. In such cases, various chemical agents such as Evista^®^ (raloxifene, focused on hormone deficiency), Fosamax^®^ (alendronate, an anti-resorption agent against osteoclasts), and Prolia^®^ (denosumab; focused on the biological process of osteoporosis) were commercially available as anti-osteoporotic agents. However, these medicines have limitations in terms of dosage and frequency of use due to their adverse effects [5]. 

Medicinal plants have been broadly used as alternative therapies in East Asia for various diseases because of their few side effects [6]. Numerous studies have demonstrated that single bioactive compounds isolated from herbal plants have beneficial effects on various symptoms such as neurodegeneration, gastrointestinal disorders, inflammation, type 2 diabetes mellitus, and obesity [7,8,9,10,11]. *Cornus officinalis* (CO) has been widely utilized as a traditional medicine, with positive effects on type 2 diabetes, liver disease, and the menopausal syndrome [12,13,14]. A previous study reported that CO inhibited osteoclast differentiation, indicating that bioactive compound(s) isolated from CO may be a potential candidate(s) responsible for the anti-osteoporotic effect [15]. Ethnopharmacological studies have demonstrated that CO consists of several bioactive components, such as loganin, morroniside, ursolic acid, and oleanolic acid, which exert multifactorial effects on various diseases [16]. Morroniside is one of the main components of the CO extract that exhibits anti-inflammatory, antioxidative, and anti-diabetic effects [17]. Previous studies have demonstrated that morroniside improves osteogenesis in the mouse preosteoblast MC3T3-E1 by activating the PI3K/Akt/mTOR signaling pathway [18]. 

However, the specific anti-osteoporotic effects of morroniside on murine models of osteoclasts and osteoporosis have not been reported. In this study, we examined the anti-osteoporotic effect of morroniside on mouse preosteoblast MC3T3-E1 cells and mouse primary cultured osteoblasts and osteoclasts in vitro and on ovariectomy (OVX)-induced mouse osteoporosis in vivo.

## 2. Results and Discussion

### 2.1. Morroniside Elevated Osteogenic Differentiation in MC3T3-E1 Cells and Mouse Primary Cultured Osteoblasts 

**Figure 1:**
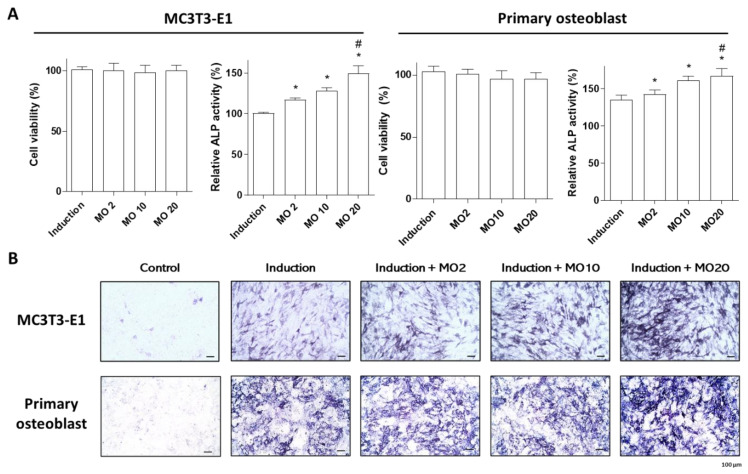
The effects of morroniside treatment in mouse preosteoblast MC3T3-E1 and mouse primary osteoblasts. Osteoblast differentiation in mouse preosteoblast MC3T3-E1 cells and mouse primary osteoblasts was induced with different concentrations of morroniside (2, 10, and 20 μM) for 3 days. (**A**) Water-soluble tetrazolium (WST) assay (left panel) and ALP activity (right panel) of morroniside treatment in MC3T3-E1 cells and mouse primary osteoblasts. (**B**) Representative images of ALP-positive cells were stained and visualized by light microscope. MO; morroniside treatment. * *p* < 0.05 vs. Induction, ^#^ *p* < 0.05 vs. MO2. (one-way ANOVA with Tukey’s honest significant difference post hoc test).

Previous studies have demonstrated that morroniside enhances osteoblast differentiation in mouse preosteoblast MC3T3-E1 cells and bone marrow mesenchymal stem cells [19,20]. Preosteoblastic cell lines used for the investigation of osteoblast-associated research, including MC3T3-E1, MG-63, and Saos2, showed a similar osteoblastic phenotype, but their functional properties and response to the regulation of osteogenic factors presented heterogeneity [21]. This study examined the osteogenic effect of morroniside in primary cultured osteoblasts isolated from mouse calvaria, compared with preosteoblastic MC3T3-E1 cells in vitro. Differentiation of preosteoblasts and primary cultured cells was induced by the administration of osteoblast induction media containing β-glycerophosphate (10 mM) and ascorbic acid (50 μg/mL) and co-treated with different concentrations of morroniside (2, 10, and 20 μM). Osteoblastogenesis was assessed by alkaline phosphatase (ALP) activity and staining. ALP is a membrane-anchored enzyme in mature osteoblasts that is expressed in large amounts of active osteoblasts involved in bone mineralization [22]. Morroniside treatment did not alter cell proliferation in either MC3T3-E1 or primary osteoblasts, indicating no cytotoxic effects (Figure 1A). However, significantly increased ALP activity and positively stained cells were observed after treatment with morroniside in a dose-dependent manner (Figure 1A and B). These results suggest that morroniside promotes osteoblast differentiation in both MC3T3-E1 cell lines and primary cultured osteoblasts.

**Figure 2:**
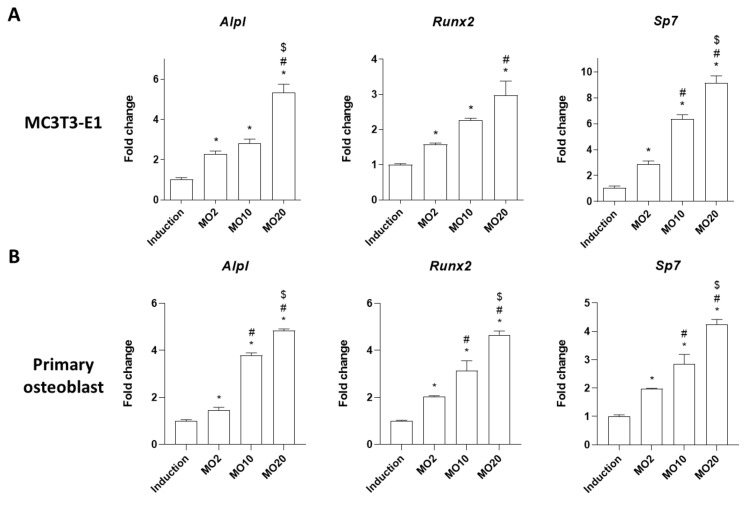
The effects of morroniside on osteoblastogenesis-specific genes in MC3T3-E1 and mouse primary osteoblasts. (**A**) MC3T3-E1 and (**B**) mouse primary osteoblasts were induced osteoblastogenesis with co-treatment of morroniside (2, 10, and 20 μM) for 3 days. mRNA expression levels of *Alpl*, *Runx2*, and *Sp7* were evaluated by qRT-PCR using specific primers. Relative expression levels were normalized by mouse *Gapdh*. MO; morroniside treatment. * *p* < 0.05 vs. Induction, ^#^ *p* < 0.05 vs. MO2, ^$^ *p* < 0.05 vs. MO20 (one-way ANOVA with Tukey’s honest significant difference post hoc test).

We further determined the effects of morroniside on mRNA expressions of osteoblastogenesis-associated genes such as *Alpl* (alkaline phosphatase, ALP), Runt-related transcription factor 2 (*Runx2*), and osterix (*Sp7*), as previously described [23]. *Runx2* is a major transcription factor involved in osteoblast differentiation [24]. *Sp7* is an osteoblast differentiation-specific gene that is downstream of *Runx2* [25]. These genes are important bone remodeling biomarkers in the regulation of osteoblast differentiation and bone formation [23,26]. MC3T3-E1 cells and mouse primary osteoblasts were treated with different concentrations of morroniside (2, 10, and 20 μM) in osteoblast induction media for 3 days, and the mRNA expression of osteoblastogenesis-associated genes was analyzed by quantitative reverse transcriptase PCR (qRT-PCR). Morroniside treatment increased the mRNA expressions of *Alpl*, *Runx2*, and *Sp7,* compared to the non-treated group (Figure 2), indicating that morroniside promoted osteoblastic differentiation by upregulating osteoblastogenesis-associated genes in both the preosteoblast MC3T3-E1 cell line and mouse primary cultured osteoblasts.

### 2.2. Morroniside Prevented Osteoclast Differentiation in Mouse Primary Monocytes

Bone remodeling is regulated by the balance between osteoclasts (bone resorption) and osteoblasts (bone formation) [1]. Deficits in osteoclast differentiation are one of the major causes of skeletal diseases, such as osteoporosis [27,28]. This study examined the anti-osteoporotic effects of morroniside on osteoclast differentiation. Numerous studies have suggested that inhibition of osteoclast differentiation or activity is a major target for the treatment of osteoporosis [29,30,31]. Osteoclasts are derived from monocyte lineage cells and osteoclast differentiation is induced by the presence of two factors, macrophage colony-stimulating factor (M-CSF) and receptor activator of nuclear kappa B ligand (RANKL) [32]. Tartrate-resistant acid phosphatase (TRAP) levels are increased by the initiation of osteoclast differentiation, leading to osteoclast migration to bone resorption sites [33]. 

**Figure 3:**
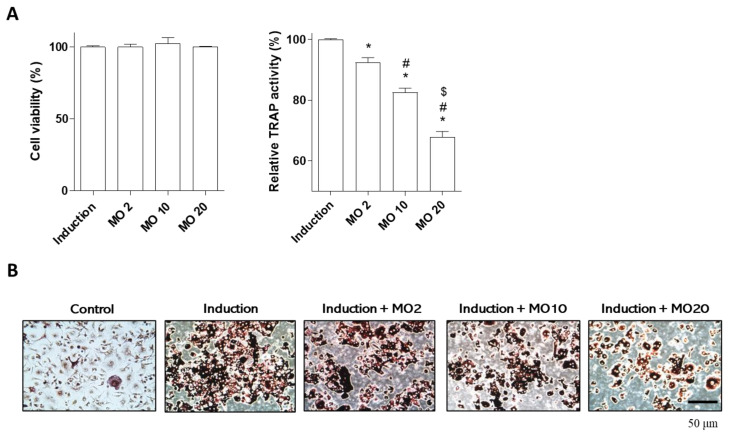
The effects of morroniside on osteoclast differentiation. Osteoclast differentiation was induced in mouse primary monocytes and they were co-treated with different concentrations of morroniside (2, 10, and 20 μM) for 5 days. (**A**) WST assay (left panel) and TRAP activity (right panel) of morroniside treatment in mouse primary osteoclasts. (**B**) Representative TRAP-positive cells were stained and visualized by a light microscope. MO; morroniside treatment. * *p* < 0.05 vs. Induction, ^#^ *p* < 0.05 vs. MO2, ^$^ *p* < 0.05 vs. MO10. (one-way ANOVA with Tukey’s honest significant difference post hoc test).

To evaluate the effect of morroniside on osteoclast differentiation, we isolated mouse primary monocytes from mouse femoral bone. Osteoclast differentiation was induced by adding osteoclast media containing M-CSF and RANKL, and co-treatment with morroniside at different concentrations (2, 10, and 20 μM) for 5 days. Morroniside did not affect the viability of primary monocytes (Figure 3A). However, morroniside treatment significantly inhibited TRAP activity during osteoclast differentiation (Figure 3A). In addition, the number of TRAP-stained multinucleated positive cells was decreased by morroniside treatment (Figure 3B). These results indicate that morroniside prevents osteoclast differentiation by inhibiting the activity of TRAP.

**Figure 4:**
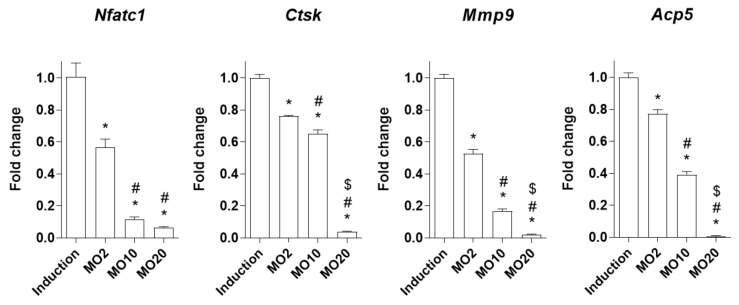
The effects of morroniside treatment on osteoclast-specific genes in mouse primary osteoclasts. Osteoclast differentiation was induced in mouse primary monocytes by co-treatment with different concentrations of morroniside (2, 10, and 20 μM) for 5 days. mRNA expression levels of *Nfatc1*, *Ctsk*, *Mmp9*, and *Acp5* were determined by qRT-PCR using gene-specific primers. The expression levels were normalized by mouse *Hprt* mRNA expression. MO; morronide treatment. * *p* < 0.05 vs. Induction, ^#^ *p* < 0.05 vs. MO2, ^$^ *p* < 0.05 vs. MO10 (one-way ANOVA followed by Tukey’s honest significant difference post hoc test).

Bone resorption (osteoclast differentiation) is regulated by several osteoclast-inducible enzymes, such as cathepsin K (*Ctsk*), matrix metalloproteinase 9 (*Mmp9*), and tartrate-resistant acid phosphatase 5 (*Acp5*) [34]. These enzymes are regulated by the nuclear factor of activated T cells 1 (*Nfatc1*), which are master regulators of osteoclastic function [35]. We investigated the inhibitory effects of morroniside on osteoclastogenic genes (*Nfatc1*, *Ctsk*, *Mmp9*, and *Acp5*), and mRNA expression levels were assessed by RT-PCR. As a result, morroniside treatment significantly decreased the mRNA expression levels of osteoclastogenic genes, including *Nfatc1*, *Ctsk*, *Mmp9*, and *Acp5,* compared to the non-treated group (Figure 4). These results indicate that morroniside prevents osteoclast activity via the downregulation of osteoclast-mediated genes associated with bone resorption.

### 2.3. Oral Administration of Morroniside Attenuated Ovariectomy (OVX)-Induced Mouse Osteoporosis

**Figure 5:**
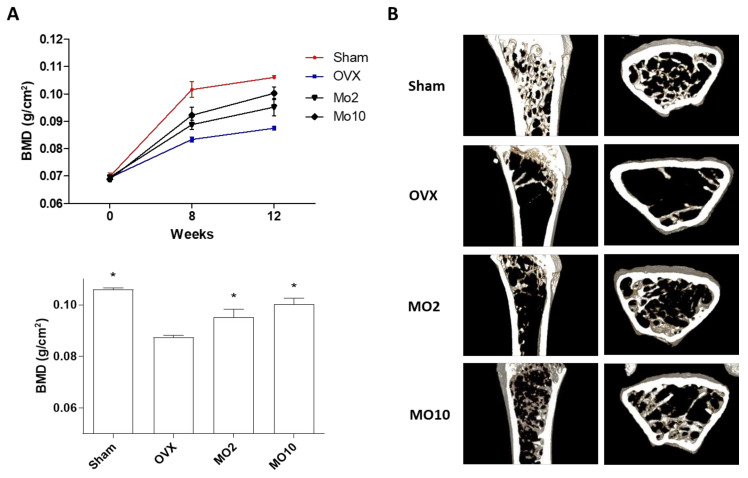
The effects of morroniside administration on an OVX-induced osteoporosis mouse model. Mice received different concentrations of morroniside (2 and 10 mg/kg/day) for 12 weeks. (**A**) Bone mineral density of right femur was determined using PIXI-mus bone densitometer during the experiment. (**B**) Representative images of right femur were obtained using Micro-CT analysis. Sham; sham-operated, OVX; ovariectomized mice, MO; morroniside treatment. ^*^ *p* < 0.05 vs. OVX (one-way ANOVA with Tukey’s honest significant difference post hoc test).

Based on the effects of morroniside in osteoblasts and osteoclasts in vitro, we examined the anti-osteoporotic effects of morroniside in ovariectomized (OVX) mice. OVX mice are a well-known murine model for evaluating skeletal effects because of their hormone deficiency, which is typically used as a postmenopausal osteoporosis model [36]. The major characteristics of OVX mice include decreased bone mineral density (BMD) of the femoral bone, which is vulnerable to fracture [37]. To determine the effects of morroniside in an osteoporotic mouse model, OVX mice were administered different concentrations of morroniside (2 or 10 mg/kg/day) for 12 weeks. This study conducted the optimal dose of in vivo experiment using the 2 to 10 mg/kg/day concentrations, based on previous study [38]. Food intake and body weight did not differ between the control group and morroniside-administered group for 12 weeks of the experiment, indicating no toxic effects (data not shown). BMD of the right femoral bone was monitored during the experiment (0, 8, and 12 weeks), and images of transverse micro-CT in the mouse right femur were obtained at the end of treatment. As expected, OVX mice showed a significant decrease in BMD with trabecular bone loss. However, morroniside administration in OVX mice prevented OVX-induced BMD loss (Figure 5A) and reduced bone structural compartment loss in micro-CT analysis of longitudinal and transverse section images (Figure 5B). These results indicated that morroniside inhibited OVX-induced osteoporotic pathogenesis in mice.

## 3. Materials and Methods

### 3.1. Cell Culture

The mouse pre-osteoblastic cell line MC3T3-E1 was obtained from the RIKEN Cell Bank (Tsukuba, Japan) and the cells were maintained in a growth medium (α-modified minimal essential medium (α-MEM, Gibco, Rockville, MD, USA) supplemented with 10% fetal bovine serum (Gibco) and 1% antibiotic-antimycotic reagent (Gibco)). Primary osteoblasts were isolated from mouse calvaria as previously described [39]. Briefly, 4-5 pup ICR mice were sacrificed by decapitation, and the calvariae were dissected from the mice. The calvariae were trimmed and subjected to collagenase II (Sigma, St. Louis, MO, USA) for 2 h at 37 °C. The first digestion was discarded, and the second digestion was neutralized with a growth medium followed by filtration using a Falcon^®^ 40 μm cell strainer. Mouse primary monocytes were isolated from bone marrow cells in the femur of a nine-week-old mouse as previously described [40]. Isolated primary monocytes were maintained in the growth medium containing 50 ng/mL of macrophage-colony stimulating factor (M-CSF; PeproTech, Cranbury, NJ, USA). All cells were maintained at 37 °C in a 5% CO_2_ incubator.

### 3.2. Osteoblast and Osteoclast Differentiation

For osteoblast differentiation, MC3T3-E1 cells and primary osteoblasts were incubated with a growth medium containing 10 mM β-glycerophosphate and 50 μg/mL ascorbic acid and co-treated with different concentrations of morroniside (2, 10, and 20 μM) without changing of the medium for 3 days. For osteoclast differentiation, primary monocytes were incubated with α-MEM containing 50 ng/mL M-CSF (PeproTech, Cranbury, NJ, USA) and 50 ng/mL RANKL (PeproTech) for 5 days. During the osteoclast differentiation period, the induction medium was changed one time at 3 days of the experiment.

### 3.3. Cell Viability

Cells were seeded in 96-well plates for 24 h and treated with morroniside for 3 days (MC3T3-E1 cells and primary osteoblasts) or 5 days (primary osteoclasts). Cell viability was assessed using the D-Plus™ CCK Cell Viability Assay Kit (Donginbiotech, Seoul, Korea). Cells were incubated with 10 μL of WST solution for 2 h, and the cell viability was measured at an absorbance of 450 nm using a microplate reader (Bio-Rad, Hercules, CA, USA).

### 3.4. ALP/TRAP Activity and Staining

Cells were harvested with cell lysis buffer (0.5 M Tris-HCL, pH 8.8, containing 0.9% NaCl, 1% Triton X-100, and 200 mM EDTA), and the ALP activity was assessed using 1-Step™ p-nitrophenylphosphate (Sigma, St. Louis, MO, USA) in accordance with the manufacturer’s instructions. For ALP staining, cells were fixed with 4% paraformaldehyde for 15 min and incubated with BCIP/NBT (Sigma) for 30 min at room temperature. TRAP activity/staining in primary osteoclasts was assessed using an Acid-Phosphatase Kit (Sigma) according to the manufacturer’s instructions. Representative images of ALP/TRAP-positive cells were visualized using a light microscope (Leica Microsystems, Wetzlar, Germany).

### 3.5. Quantitative Reverse Transcriptase Polymerase Chain Reaction (qRT-PCR) Analysis

Total RNA was harvested from cultured cells using TRIzol reagent (Invitrogen, Carlsbad, CA, USA) according to the manufacturer’s instructions. Complementary DNA (cDNA) was synthesized using the RevertAid™ H Minus First Strand cDNA Synthesis Kit (Fermentas, Hanover, NH, USA). qRT-PCR was performed using the SYBR Green I qPCR Kit (TaKaRa, Shiga, Japan) with gene-specific primers. The specific primer sequences were as follows: forward 5′-TCC CAC GTT TTC ACA TTC GG-3′ and reverse 5′-CCC GTT ACC ATA TAG GAT AGC C-3′ for mouse *Alpl*, forward 5′-TAA AGT GAC AGT GGA CGG TCC C-3′ and reverse 5′- AAT GCG CCC TAA ATC ACT GAG G-3′ for mouse *Runx2*, forward 5′-CAG GAA GAA GCT CAC TAT GG-3′ and reverse 5′-GTC CAT TGG TGC TTG AGA AG-3′ for mouse *Sp7*, forward 5′-GGA GAG TCC GAG AAT CGA GAT-3′ and reverse 5′-TTG CAG CTA GGA AGT ACG TCT-3′ for mouse *Nfatc1*, forward 5′-AAT ACC TCC CTC TCG ATC CTA CA-3′ and reverse 5′-TGG TTC TTG ACT GGA GTA ACG TA-3′ for mouse *Ctsk*, forward 5′-CTT CGA CAC TGA CAA GAA GTG G-3′ and reverse 5′-GGC ACG CTG GAA TGA TCT AAG-3′ for mouse *Mmp9*, forward 5′-TGG TAT GTG CTG GCT GGA AAC-3′ and reverse 5′-AGT TGC CAC ACA GCA TCA CTG-3′ for mouse *Acp5*, forward 5′-AGG TCG GTG TGA ACG GAT TTG-3′ and reverse 5′-TGT AGA CCA TGT AGT TGA GGT CA-3′ for mouse *Gapdh*, forward 5′-GAG GAG TCC TGT TGA TGT TGC CAG-3′ and reverse 5′-GGC TGG CCT ATA GGC TCA TAG TGC-3′ for mouse *Hprt*. Gene expression levels were normalized to mouse *Gapdh* (osteoblast) or *Hprt* (osteoclast) expression, and the results were presented as the 2^-ΔΔCt^ method (ΔΔCt = ΔCt_Treatment_-ΔCt-_Induction_).

### 3.6. Animal Study

Eight-week-old sham-operated or OVX ddY mice were obtained from Shizuoka Laboratory Center, Inc. (Hamamatsu, Japan). Mice were housed under controlled pathogen-free conditions at the Laboratory Animal Research Center of Ajou University Medical Center, provided with sterile food pellets (Teklad-certified irradiated global 18% protein rodent diet, Harlan Teklad, Madison, WI, USA) and sterile water ad libitum. For the experiment, mice were administered different concentrations of morroniside (2 or 10 mg/kg/day) for 12 weeks. All animal experiments, including primary cell culture, were approved by the Institutional Animal Care and Use Committee (IACUC) of Ajou University School of Medicine (2016-0062).

### 3.7. Bone Mineral Density (BMD) and micro-CT Analysis

Mice were anesthetized with zolazepam/tiletamine (Zoletil; Virbac Laboratories, Carros, France) by intraperitoneal injection, and the BMD was measured using a PIXI-mus bone densitometer (GE Lunar, Madison, WI, USA). At the end of the experiment, mice were sacrificed, and the right femur was fixed in 4% paraformaldehyde for 24 h, and then placed on the sample holder of the scanner. The scans were performed along the longitudinal axis of the specimen using a Skyscan 1173 (Bruker, Kontich, Belgium) under identical conditions (400 μA, 60 kV, charge-coupled device readout of 1280 × 1280, 400 ms exposure time, rotation steps of 360°). Three-dimensional axial images were reconstructed, and representative two-dimensional images were captured using the NRecon software (Bruker microCT, North Billerica, MA, USA).

### 3.8. Statistical Analysis

Data in the bar graphs are expressed as the mean ± standard error of the mean (S.E.M.) using GraphPad Prism 9.0 software (GraphPad Software, San Diego, CA, USA). Comparisons of multiple groups were analyzed by one-way analysis of variance (ANOVA) with Tukey’s honest significant difference (HSD) post-hoc test. *p* < 0.05 was considered as statistically significant.

## 4. Conclusions

In the present study, we examined the anti-osteoporotic effect of morroniside in osteoblasts and osteoclasts in vitro and in an OVX-induced osteoporosis mouse model in vivo. Morroniside promoted osteoblast differentiation by upregulating ALP activity and osteoblastogenesis-associated genes. By contrast, morroniside prevented osteoclastogenic differentiation by inhibiting TRAP activity and the expression of osteoclastogenic genes. In a murine osteoporotic model, morroniside administration prevented OVX-induced BMD loss and bone mineral compartment in the femoral bone. Taken together, these results indicate that morroniside may be a potential therapeutic single compound for the prevention and treatment of osteoporosis pathogenesis by improving bone homeostasis.

## Data Availability

The data presented in this study are available on request from the corresponding author.
